# Optimization, Stability, and Entropy in Endoreversible Heat Engines

**DOI:** 10.3390/e22111323

**Published:** 2020-11-20

**Authors:** Julian Gonzalez-Ayala, José Miguel Mateos Roco, Alejandro Medina, Antonio Calvo Hernández

**Affiliations:** 1 Instituto de Física Fundamental y Matemáticas, Universidad de Salamanca, 37008 Salamanca, Spain; roco@usal.es (J.M.M.R.); amd385@usal.es (A.M.); anca@usal.es (A.C.H.); 2 Departamento de Física Aplicada, Universidad de Salamanca, 37008 Salamanca, Spain

**Keywords:** multiobjective optimization, Pareto front, stability, maximum power regime, entropy behavior

## Abstract

The stability of endoreversible heat engines has been extensively studied in the literature. In this paper, an alternative dynamic equations system was obtained by using restitution forces that bring the system back to the stationary state. The departing point is the assumption that the system has a stationary fixed point, along with a Taylor expansion in the first order of the input/output heat fluxes, without further specifications regarding the properties of the working fluid or the heat device specifications. Specific cases of the Newton and the phenomenological heat transfer laws in a Carnot-like heat engine model were analyzed. It was shown that the evolution of the trajectories toward the stationary state have relevant consequences on the performance of the system. A major role was played by the symmetries/asymmetries of the conductance ratio $\sigma_{hc}$ of the heat transfer law associated with the input/output heat exchanges. Accordingly, three main behaviors were observed: (1) For small $\sigma_{hc}$ values, the thermodynamic trajectories evolved near the endoreversible limit, improving the efficiency and power output values with a decrease in entropy generation; (2) for large $\sigma_{hc}$ values, the thermodynamic trajectories evolved either near the Pareto front or near the endoreversible limit, and in both cases, they improved the efficiency and power values with a decrease in entropy generation; (3) for the symmetric case ($\sigma_{hc}=1$), the trajectories evolved either with increasing entropy generation tending toward the Pareto front or with a decrease in entropy generation tending toward the endoreversible limit. Moreover, it was shown that the total entropy generation can define a time scale for both the operation cycle time and the relaxation characteristic time.

## 1. Introduction

The optimization of energy converters has never been as relevant as it is now. Energy production requirements, efficient use of heat sources, and heat waste reduction are continually pushing technological edges. Linked to this degree of specialization are the control and stability of operation regimes yielding a desirable stable production, despite the possible variations of external or internal conditions. In many cases, this requires the fine-tuning of control parameters with intrinsic energetic costs. In this regard, there are some hints as to the possibility of seizing the stability of heat engine operation regimes to enhance their performance by relaxing the control over the operation parameters [1,2,3]. Studies regarding the weakly dissipative limit [4] thus far have pointed out the special role played by the endoreversible model [5,6]. These studies consider that after the system experiences a perturbation in its operation variables, the trajectories that lead the system back to its steady state tend to evolve toward the endoreversible limit. This limit has been proven to be associated with thermodynamic states, with the best compromise between maximum efficiency, maximum power output, and minimum entropy production as given by the Pareto front of the system. Although the equivalence of the low dissipation model (based on entropy generation) and the endoreversible model (based on specific heat transfer laws) has been established for several heat transfer laws [7,8,9], it is not obvious that the endoreversible behavior appears interconnected to the stability of the low dissipation model. A natural concern is to look for this very same behavior in the context of endoreversible heat engines.

On the other side, the stability of heat engines is not a novel topic. Especially in the realm of finite-time thermodynamics, endoreversible and irreversible models have, in their assets, a good number of works in this regard. From the pioneering work of Santillan et al. [10], a number of studies have analyzed the local and global stability of a variety of operation regimes [11,12,13,14,15,16,17,18], including economic factors [19], and have extended the analysis to heat pumps, refrigerators, and generalized heat engines [17,20,21,22,23,24,25,26,27].

The validation and applicability of the endoreversible hypothesis has been widely analyzed and discussed in the specialized literature [28,29,30,31], and although it constitutes an idealization of an irreversible device, recent studies on molecular dynamics simulation [32] have validated its predictions for a finite-time Carnot cycle of a weakly interacting gas (considered as a nearly ideal gas) and considering the local equilibrium for a Maxwell–Boltzmann distribution with a spatially uniform temperature and a spatially varying local center-of-mass velocity. In particular, these results also point to the validity of the paradigmatic Curzon–Ahlborn efficiency at maximum power [33,34]. These molecular dynamics simulations of a two-dimensional Carnot engine allowed to investigate not only the optimization of power output, but also some other figures of merit involving entropy production as the ecological figure of merit [32]. This reinforces the validity of the Carnot-like endoreversible model, where the quasistatic conditions linked to endoreversibility rely on the thermalization due to the internal dynamic speed. Lastly, a recent work reported some new conceptual insights (simulation and reconstruction) on the endoreversibility hypothesis [35] by considering that subsystems are out of equilibrium, i.e., including internal irreversibilities: First, by means of the contact temperature of the heat flows and the non-equilibrium molar entropy for material flows. This new feature is beyond the traditional endoreversible thermodynamics, which considers internal subsystems as reversible, i.e., without internal entropy production. Second, the mentioned work [35] goes beyond the use of paradigmatic, simple models and thus sheds light on the modeling characteristics of endoreversible systems in relation to real running heat engines.

Despite the extensive literature, the possibility of inducing optimization from stability in finite-time thermodynamics has not been studied. This is one of the goals of the present paper, which is focused on irreversible Carnot-like heat engine models with linear and inverse heat transfer laws, as two representative examples, assuming that the maximum power state behaves as a steady state. The kind of perturbations assumed in this analysis are from external sources or variations in the external control, such as those stemming from variations in the velocity of a piston. A benefit of this study is that, unlike the low-dissipation scheme, based on entropy production, the finite-time thermodynamics scheme allows a more straightforward analysis of its consequences on the working fluid and design of the heat engine, as it accounts for explicit heat transfer laws.

This paper is structured as follows: In Section 2, an overview of the endoreversible model is presented, along with some results on the maximum power regime for both the Newton and the phenomenological heat transfer laws. In Section 3, a multiobjective optimization is realized and the Pareto front is faced with endoreversible behavior. In Section 4, the stability dynamics are obtained from basic assumptions and the operation and relaxation times are compared in order to achieve a useful stability. In Section 5, the relaxation trajectories are analyzed in the space of the control variables as in the energetic space composed by power output, efficiency, and entropy generation. Finally some concluding remarks are presented.

## 2. A Quick Look at the Endoreversible Model

**Figure 1:**
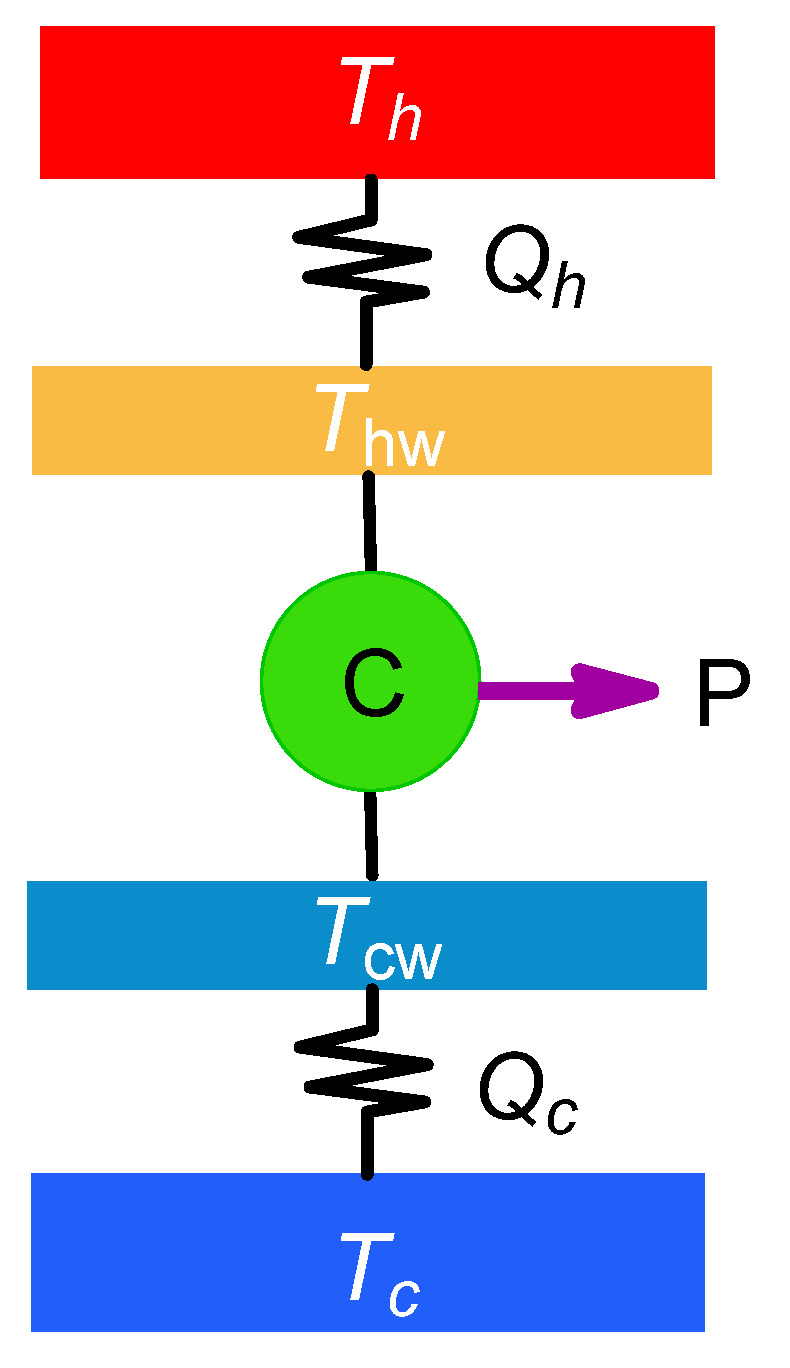
Schematic representation of an endoreversible heat engine. The working fluid realizes a Carnot cycle operating between the isothermal processes at effective temperatures $T_{hw}$ and $T_{cw}<T_{hw}$. The working fluid is irreversibly coupled to external reservoirs at temperatures $T_{h}$ and $T_{c}<T_{h}$.

The most basic endoreversible model consists on a baseline Carnot cycle whose working fluid operates with effective temperatures $T_{hw}$ and $T_{cw}$ (isotherms of the Carnot cycle), irreversibly connected to two external reservoirs at temperature $T_{h}$ and $T_{c}$ ($T_{h}>T_{hw}>T_{cw}>T_{c}$), as depicted in Figure 1.

The endoreversible scheme requires knowledge of the heat transfer laws to model the heat fluxes between the external reservoirs and the working fluid. In a somewhat general case of heat transfer laws, $Q_{h}$ and $Q_{c}$ are expressed as:

(1) $$\begin{array}{ccc} Q_{h} & = & \sigma_{hc}\sigma_{c}\left(T_{h}^{k}-T_{hw}^{k}\right)sgn\left(k\right)t_{h}>0, \end{array}$$

(2) $$\begin{array}{ccc} Q_{c} & = & \sigma_{c}\left(T_{c}^{k}-T_{cw}^{k}\right)sgn\left(k\right)t_{c}<0, \end{array}$$

where k≠0 is the exponent of the heat transfer law (k=1 refers to the known Newton law, k=−1 is known as the phenomenological law, k=4 is the Stefan–Boltzmann law, and so on); $\sigma_{c}$ and $\sigma_{h}$ are the thermal conductances with units of J·K${}^{-k}/$s, here considered as constant since only small fluctuations around the operation regime are addressed. The function $sgn\left(k\right)$ is defined as:

$$sgn\left(k\right)=\left\{\begin{array}{cc} 1 & if\;k>0\\ -1 & if\;k<0 \end{array}\right.,$$

which is introduced for consistency with the convention that the heat flux entering the working fluid is positive and that the outgoing heat is negative; $t_{h}$ and $t_{c}$ are the times at which the working fluid is in contact with the external heat reservoirs $T_{h}$ and $T_{c}$, respectively. In Equation (1), the $\sigma_{hc}\equiv \sigma_{h}/\sigma_{c}$ ratio is introduced, which is an important ingredient to obtain the upper and lower bounds of the efficiency of a given operation regime. The endoreversible hypothesis states that the entropy generation in the inner part of the engine is zero. Mathematically, this means that:

(3) $$\frac{Q_{h}}{T_{hw}}+\frac{Q_{c}}{T_{cw}}=0,$$

and from this constraint, the ratio of the operation time is given as:

(4) $$\frac{t_{c}}{t_{h}}=\frac{T_{h}^{k}-T_{hw}^{k}}{T_{cw}^{k}-\tau^{k}T_{h}^{k}}\;\frac{T_{cw}}{T_{hw}}\sigma_{hc},$$

where $\tau \equiv T_{c}/T_{h}$. With the endoreversible hypothesis constraining $t_{c}$, in Equations (3) and (4), the efficiency η, power output *P*, and total entropy generation *S* become functions of {$T_{h}$, $T_{c}$, $T_{hw}$, $T_{cw}$, $\sigma_{hc}$, $t_{h}$, $\sigma_{c}$}. By using Equations (1) and (2), the explicit equations of these key thermodynamic magnitudes are the following:

(5) $$\begin{array}{c} \begin{array}{c} \eta =1+\frac{Q_{c}}{Q_{h}}=\begin{array}{cc} 1-\frac{T_{cw}}{T_{hw}}, & k=\{1,-1\} \end{array}\\ P=\frac{Q_{h}+Q_{c}}{t_{c}+t_{h}}=\left\{\begin{array}{cc} \frac{\sigma_{c}\sigma_{hc}\left(T_{cw}-T_{hw}\right)\left(T_{h}-T_{hw}\right)\left(T_{cw}-\tau T_{h}\right)sgn\left(k\right)}{T_{cw}T_{hw}\left(\sigma_{hc}-1\right)+T_{h}\left(\tau T_{hw}-\sigma_{hc}T_{cw}\right)} & k=1\\ \frac{\sigma_{c}\sigma_{hc}\left(T_{cw}-T_{hw}\right)\left(T_{h}-T_{hw}\right)\left(T_{cw}-\tau T_{h}\right)sgn\left(k\right)}{T_{h}\left(T_{hw}^{2}\left(T_{cw}-\tau T_{h}\right)+T_{cw}^{2}\left(T_{h}-T_{hw}\right)\tau \sigma_{hc}\right)} & k=-1 \end{array}\right.,\\ S=-\frac{Q_{h}}{T_{h}}-\frac{Q_{c}}{T_{c}}=\left\{\begin{array}{cc} \frac{t_{h}\sigma_{c}\sigma_{hc}\left(T_{h}-T_{hw}\right)\left(T_{cw}-\tau T_{hw}\right)sgn\left(k\right)}{T_{h}T_{hw}\tau } & k=1\\ \frac{t_{h}\sigma_{c}\sigma_{hc}\left(T_{h}-T_{hw}\right)\left(\tau T_{hw}-T_{cw}\right)sgn\left(k\right)}{T_{h}^{2}T_{hw}^{2}\tau } & k=-1 \end{array}\right.. \end{array} \end{array}$$

As can be seen in the definition of the power output, instantaneous adiabatic processes were considered. In order to deal with non-instantaneous adiabatics, a more detailed analysis of the working fluid and the geometry of the device needs to be made (for example, see [36]). In some works, these features have been analyzed by showing their influence in the power output and efficiency, but under certain circumstances, such as the large compression ratios in a piston, these times produce small effects on η and *P*, remaining compatible with the quasistatic nature of the internal process.

Notice that only *S* is proportional to $t_{h}$. As is shown later in Section 4 (see Equation (15)), the relaxation times are also proportional to this partial time; thus, a relationship between relaxation time and total entropy generation can be found. By fixing the value of *S*, a time scale for stability can be established. Besides this time scale (establishing the speed of the restitution dynamics), it is possible to analyze the energetic consequences of stability independently of $t_{h}$ if one works with entropy production per cycle time, defined as $\dot{S}\equiv S/\left(t_{c}+t_{h}\right)=S/t_{h}\left(1+t_{c}/t_{h}\right)$, taking advantage of the constraint on the total operation time through Equation (4).

The maximum power (MP) regime is specified by the temperatures $T_{cw}^{*}$ resulting from the constraint $\partial P/\partial T_{cw}=0$ and $T_{hw}^{*}$ under the condition $\partial P/\partial T_{hw}=0$. The resulting values are given by:

(6) $$\begin{array}{ccc} T_{hw}^{*} & =\frac{T_{h}\left(\sqrt{\tau }+\sqrt{\sigma_{hc}}\right)}{1+\sqrt{\sigma_{hc}}} & k=1, \end{array}$$

(7) $$\begin{array}{ccc} T_{cw}^{*} & =\frac{T_{h}\left(\tau +\sqrt{\sigma_{hc}\tau }\right)}{1+\sqrt{\sigma_{hc}}} & k=1, \end{array}$$

(8) $$\begin{array}{ccc} T_{hw}^{*} & =\frac{2T_{h}\tau \left(1+\sqrt{\sigma_{hc}}\right)}{1+\tau +2\tau \sqrt{\sigma_{hc}}} & k=-1, \end{array}$$

(9) $$\begin{array}{ccc} T_{cw}^{*} & =\frac{2T_{h}\tau \left(1+\sqrt{\sigma_{hc}}\right)}{2+\sqrt{\sigma_{hc}}\left(1+\tau \right)} & k=-1. \end{array}$$

**Figure 2:**
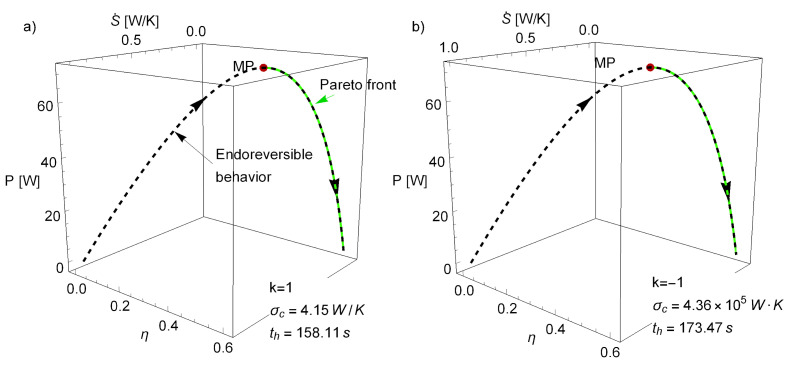
Parabolic behavior of the η, *P*, and $\dot{S}$ curve typical of the endoreversible model for (**a**) the k=1 case and (**b**) the k=−1 case. The values $T_{h}$ = 500 K, $\sigma_{hc}=1$, and τ=0.4 weer fixed. Additionally, $\sigma_{c}$ was chosen (for representation/comparison purposes) in such a way that the maximum power (MP) was 70 W in both cases, so that η, *P*, and $\dot{S}$ ranged in similar intervals.

From the optimal $T_{cw}^{*}$ values, parametrization of *P*, η, and $\dot{S}$ can be made (see dashed curve in Figure 2). The obtained parametric curve exhibits a parabolic-like behavior, where the values of η vary from 0 to $\eta_{C}\equiv 1-\tau$, the Carnot efficiency (both limits corresponding to a zero power output), and with a single maximum in *P*. This is what is known in the literature as an endoreversible behavior, a kind of signature of the model. Some relevant features regarding the efficiency at maximum power from these endoreversible models with k=1 and k=−1 are pointed out:

- The Newton case (k=1) gives an efficiency at maximum power, $\eta_{MP}=\eta_{CAN}\equiv 1-\sqrt{\tau }$, the well-known Curzon–Ahlborn–Novikov (CAN) efficiency, which does not depend on the $\sigma_{hc}$ ratio and appears in a large variety of contexts linked to the maximization of power output, work, and kinetic energy [37].
- The k=−1 case, frequently called the phenomenological law, referring to the natural results arising in the linear irreversible thermodynamics framework. It allows to obtain the same limits of efficiency as in the low-dissipation model, where the self-optimization property has been studied. In this case (k=−1), $\eta_{MP}$ is $\sigma_{hc}$-dependent, bounded by $\eta_{MP}\in \left(\frac{\eta_{C}}{2},\frac{\eta_{C}}{2-\eta_{C}}\right)$, according to whether $\sigma_{hc}$ varies from 0 to *∞* [7].

## 3. The Relevant Region for Optimization: The Pareto Front

Before exploring stability dynamics, it is convenient to introduce a multiobjective optimization of the model. When looking for the best compromise among a variety of objective functions, the result is the so-called Pareto front (in the space of energetic functions) and the corresponding Pareto optimal set (in the space of operation variables).

To this end, a sorting algorithm is used by applying the concept of dominance [38]: A vector $v=(v_{1},\ldots ,v_{n})$ dominates another one $w=(w_{1},\ldots ,w_{n})$ if, and only if, $v_{i}\geq w_{i}\;\forall \;i\in \left\{1,\ldots ,n\right\}$ (if one is looking for a maximum, ≤ for a minimum) and there is at least one *j*, such that $v_{j}>w_{j}$, if one is interested in those vectors that are not dominated by any other. In other words, vectors that have the best value at least in one objective function. In this case, such a vector is formed by η, *P*, and $\dot{S}$. The algorithm introduced here is a modification of the one introduced in [1,2,3], as follows:

1. In the phase space ($T_{hw}$, $T_{cw}$), the region of physical relevance is defined ($T_{h}\geq T_{hw}\geq T_{cw}\geq T_{c}$).
2. A random set of points in the phase space is obtained and the thermodynamic functions are evaluated (energetic space).
3. A set of non-dominated points in the energetic space is obtained, giving a provisional Pareto front.
4. From the corresponding Pareto optimal set (phase space), a convex region is defined and extended in order to cover a larger region for searching new points in the Pareto front. Details on the definition of the extended region are given below.
5. From the new region, a new set of random points is proposed and a new set of non-dominated points in the energetic space is obtained.

In the present analysis, and in order to ensure convergence in the results, Kullback–Leibler (KL) divergence was introduced as a measure of the relative entropy $D_{KL}$ [39]. $D_{KL}$ was calculated between the probability distribution of the efficiencies of the Pareto front in the *i*th and the i−1th iterations. As the probability distribution of η converges to the true Pareto front distribution, the entropy of the distribution converges as well. In such a case, the relative entropy $D_{KL}$ tends toward zero. The radius to extend the search region in the phase space decreases with the $D_{KL}$ value. When this relative entropy is very small, there is no information gain in iterating more times; then, the search for new points in the Pareto optimal set stops. As mentioned before, $D_{KL}$ provides a measure of statistical convergence by indicating how distant two distributions are. If $D_{KL}=0$, then the information stemming from both distributions is the same. This is a relevant issue to demonstrate that the obtained trend is not due to the lack of additional iterations.

To obtain $D_{KL}$, the range of possible values of η, each iteration was divided into $\sqrt{N}$ (rounded to the upper next integer [40], with *N* being the number of random points added to the search in each iteration) equal intervals (or bins). In this way, the same partition was used to compute the discrete probability distributions, $\rho_{k}$ (for the *k* iteration). $D_{KL}$ was calculated by comparing $\rho_{k-1}$ with $\rho_{k}$. $D_{KL,k}$ is given by:

(10) $$D_{KL,k}\left(\rho_{k-1}∥\rho_{k}\right)=-\sum_{i}\rho_{k-1,i}\;log\left(\frac{\rho_{k,i}}{\rho_{k-1,i}}\right),$$

allowing to determine how much information is gained by narrowing the search. In Figure 2, the Pareto front is depicted (green points), along with the endoreversible curve for η, *P*, and $\dot{S}$ (dashed line), obtained from the parametric elimination of $T_{hw}$ ($T_{cw}$ is constant for the fixed values of the $\sigma_{hc}$, $\sigma_{c}$, τ, and $T_{h}$ parameters; see Equations (7) and (9)). Notice that even when the endoreversible curve depends on a first constraint ($T_{cw}$ satisfies $\partial P/\partial T_{cw}=0$), the Pareto front has nothing to do with. Nonetheless, the Pareto front lies over the endoreversible curve, covering the regions from maximum power to maximum efficiency and minimum entropy production. This is consistent with the literature, where the mentioned part of the curve has been denoted as the relevant region for optimization. For optimization purposes, additional figures of merit as compromise functions between these three (such as the Ecological function [41] or the Omega function [42]) will not provide further information to the Pareto front. After all of these considerations, analysis of stability dynamics can be made.

## 4. Stability Dynamics and Relaxation Times

One goal of this analysis was to recover the previous results obtained in the low-dissipation scheme (with no direct connection to working fluid particularities) and to lay the groundwork for a more direct connection with the properties of the working fluid and design parameters. The perturbations we were interested in involved only those of an external nature. The temperature of the external reservoirs remained constant, as well as the conductances of the heat transfer laws (lastly depending on temperature) for only small variations in the operation regime. To illustrate these points, consider a 2-D piston describing a Carnot cycle. The velocity of the piston will determine the effective temperatures of the isotherms of the particles inside the piston (see [32]). The external control (and its energetic cost) needs to remain constant as the velocity of the piston is not accounted for in the thermodynamic description of the gas inside of it. However, fluctuations in this velocity, as well as possible cyclic variability, will lead to variations in the effective temperatures $T_{hw}$ and $T_{cw}$. Then, a compromise between the energetic cost of the control and the thermodynamic consequences due to stability is of interest.

A simple approach to tackle this problem is the following: Since the operation regime is entirely defined by $T_{hw}$ and $T_{cw}$, the dynamics involving only these two variables are to be addressed. Fluctuations in these temperatures will affect the heat exchanged between the working fluid and the external reservoirs, $Q_{h}$ and $Q_{c}$. From a Taylor expansion in the first order, $Q_{h}$ and $Q_{c}$ near the MP state can be written in matrix form as:

(11) $$\begin{array}{cc} & \left(\begin{array}{c} Q_{h}-Q_{h}^{*}\\ Q_{c}-Q_{c}^{*} \end{array}\right)=\left(\begin{array}{cc} {\left.\frac{\partial Q_{h}}{\partial T_{hw}}\right|}_{*} & {\left.\frac{\partial Q_{h}}{\partial T_{cw}}\right|}_{*}\\ {\left.\frac{\partial Q_{c}}{\partial T_{hw}}\right|}_{*} & {\left.\frac{\partial Q_{c}}{\partial T_{cw}}\right|}_{*} \end{array}\right)\cdot \left(\begin{array}{c} T_{hw}-T_{hw}^{*}\\ T_{cw}-T_{cw}^{*} \end{array}\right)=\left(\begin{array}{cc} \alpha & 0\\ \beta & \gamma \end{array}\right)\cdot \left(\begin{array}{c} T_{hw}-T_{hw}^{*}\\ T_{cw}-T_{cw}^{*} \end{array}\right) \end{array}$$

(12) $$\begin{array}{cc} & \Rightarrow \;\left(\begin{array}{c} T_{hw}-T_{hw}^{*}\\ T_{cw}-T_{cw}^{*} \end{array}\right)=-\left(\begin{array}{cc} \frac{1}{\alpha } & 0\\ -\frac{\beta }{\alpha \gamma } & \frac{1}{\gamma } \end{array}\right)\cdot \left(\begin{array}{c} Q_{h}^{*}-Q_{h}\\ Q_{c}^{*}-Q_{c} \end{array}\right), \end{array}$$

where α, β, and γ are the elements of the Jacobian matrix evaluated in the MP state (denoted by ∗). It is noted again that this analysis assumes that the MP state is a steady stationary state. Within the first order scheme [43], the simplest relationship for an autonomous system for the two variables $T_{hw}$ and $T_{cw}$ is:

(13) $$\begin{array}{cc} & \left(\begin{array}{c} {\dot{T}}_{hw}\\ {\dot{T}}_{cw} \end{array}\right)=-\left(\begin{array}{cc} A & 0\\ 0 & B \end{array}\right)\cdot \left(\begin{array}{c} T_{hw}-T_{hw}^{*}\\ T_{cw}-T_{cw}^{*} \end{array}\right), \end{array}$$

which is a good approximation near a stable point. The coefficients *A* and *B* determine the restitution strength (with units of s${}^{-1}$). The bigger they are, the faster the system will return to the steady state. By substituting Equation (12) into Equation (13), with the resulting dynamics:

(14) $$\begin{array}{cc} & \left(\begin{array}{c} {\dot{T}}_{hw}\\ {\dot{T}}_{cw} \end{array}\right)=\left(\begin{array}{cc} \frac{A}{\alpha } & 0\\ -\frac{B\beta }{\alpha \gamma } & \frac{B}{\gamma } \end{array}\right)\cdot \left(\begin{array}{c} Q_{h}^{*}-Q_{h}\\ Q_{c}^{*}-Q_{c} \end{array}\right). \end{array}$$

The magnitudes of the relaxation times $t_{1}=-1/\lambda_{1}$ and $t_{2}=-1/\lambda_{2}$ are obtained from the eigenvalues, $\lambda_{1,2}$, of the square matrix in the right-hand side of Equation (14). For the cases k=1 and k=−1, they are:

(15) $$\begin{array}{c} \begin{array}{cc} t_{1}= & \left\{\begin{array}{cc} \frac{t_{h}\sigma_{c}\sigma_{hc}}{A} & k=1\\ \frac{t_{h}\sigma_{c}\sigma_{hc}\left(1+\tau +2\tau \sqrt{\sigma_{hc}}\right)}{4AT_{h}^{2}\tau^{2}{\left(1+\sqrt{\sigma_{hc}}\right)}^{2}} & k=-1, \end{array}\right.\\ t_{2}= & \left\{\begin{array}{cc} \frac{t_{h}\sigma_{c}\sigma_{hc}\left(1-\sqrt{\tau }\right)}{B\left(\sqrt{\sigma_{hc}}+\sqrt{\tau }\right)} & k=1\\ \frac{t_{h}\sigma_{c}\sigma_{hc}\left(1+\tau +2\tau \sqrt{\sigma_{hc}}\right)\left(1-\tau \right)}{4BT_{h}^{2}\tau^{2}{\left(1+\sqrt{\sigma_{hc}}\right)}^{2}} & k=-1. \end{array}\right. \end{array} \end{array}$$

Due to the units of the two matrices, a K/J unit factor should be accounted for in the final expression on the right-hand side of Equations (15), providing the correct units for the relaxation times (in s). As mentioned before, *A* and *B* provide the strength of the restitution dynamics, i.e., if they have large values, then the relaxation times are short. Notice that both the relaxation times and the total entropy generation are proportional to the contact time $t_{h}$; then, *S* can be used to define a characteristic time scale for relaxation. Since there are no reasons to provide a preferred relaxation in the heat exchange $Q_{c}$ or $Q_{h}$, it can be assumed that $t_{1}=t_{2}$ (this requirement can be tuned according to the specific conditions of a heat device at hand). Additionally, when looking for stability of a cyclic process, it is desirable that the stability is achieved in times shorter than those of the operation time; that is:

(16) $$t_{1}+t_{2}=2t_{1}\leq t_{h}+t_{c}=t_{h}\left(1+\frac{t_{c}}{t_{h}}\right).$$

From this constraint and Equation (4), the relaxation times are bounded by:

(17) $$\begin{array}{c} t_{1}=t_{2}\leq \left\{\begin{array}{cc} \frac{t_{h}}{2}\left(1+\sqrt{\sigma_{hc}}\right) & k=1\\ \frac{t_{h}\left(1+\sqrt{\sigma_{hc}}\right)\left(1+\tau \sqrt{\sigma_{hc}}\right)}{2+\sqrt{\sigma_{h}c}\left(1+\tau \right)} & k=-1, \end{array}\right. \end{array}$$

where the equality corresponds to the case where $t_{c}+t_{h}=t_{1}+t_{2}$. From now on, equality is assumed. Then, the resulting dynamics (Equation (14)) for k=1 are:

(18) $$\begin{array}{c} \begin{array}{cc} {\dot{T}}_{hw} & =-\frac{2\sigma_{c}\sigma_{hc}\left(T_{hw}\left(1+\sqrt{\sigma_{hc}}\right)-T_{h}\left(\sqrt{\sigma_{hc}}+\sqrt{\tau }\right)\right)}{{\left(1+\sqrt{\sigma_{hc}}\right)}^{2}},\\ {\dot{T}}_{cw} & =-\frac{2\sigma_{c}\sigma_{hc}T_{h}\sqrt{\tau }}{1+\sqrt{\sigma_{hc}}}\left(\frac{T_{hw}\left(1+\sqrt{\sigma_{hc}}\right)}{T_{h}\left(\sqrt{\sigma_{hc}+\sqrt{\tau }}\right)}+\frac{T_{cw}}{T_{h}\sqrt{\tau }}\left(\frac{T_{h}}{T_{hw}}-1\right)-\frac{1-\tau +2\left(\sqrt{\sigma_{hc}}+\sqrt{\tau }\right)}{\left(1+\sqrt{\sigma_{hc}}\right)\left(1+\sqrt{\tau }\right)}\right), \end{array} \end{array}$$

and for k=−1,

(19) $$\begin{array}{c} \begin{array}{cc} {\dot{T}}_{hw} & =-\frac{4\sigma_{c}\sigma_{hc}}{T_{hw}}\cdot \frac{\left(\frac{T_{hw}}{T_{h}}\left(\frac{1+\tau }{2\tau }+\sqrt{\sigma_{hc}}\right)-1-\sqrt{\sigma_{hc}}\right)\left(1+\sqrt{\sigma_{hc}}\left(\frac{1+\tau }{2}\right)\right)}{{\left(1+\sqrt{\sigma_{hc}}\right)}^{2}\left(1+\tau \sqrt{\sigma_{hc}}\right)},\\ {\dot{T}}_{cw} & =-\frac{4\sigma_{c}\sigma_{hc}}{T_{hw}}\cdot \frac{\frac{\tau T_{hw}}{T_{h}}{\left(\frac{1+\tau }{2\tau }+\sqrt{\sigma_{hc}}\right)}^{2}-\left(1+\sqrt{\sigma_{hc}}\right)\left(1+\tau \sqrt{\sigma_{hc}}+\frac{T_{cw}}{T_{h}}\left(1+\sqrt{\sigma_{hc}}\left(\frac{1+\tau }{2}\right)\right)\right)+\frac{T_{cw}}{T_{hw}}\left(1+\sqrt{\sigma_{hc}}\right)\left(1+\tau \sqrt{\sigma_{hc}}\right)}{{\left(1+\sqrt{\sigma_{hc}}\right)}^{2}\left(1+\tau \sqrt{\sigma_{hc}}\right)}. \end{array} \end{array}$$

**Figure 3:**
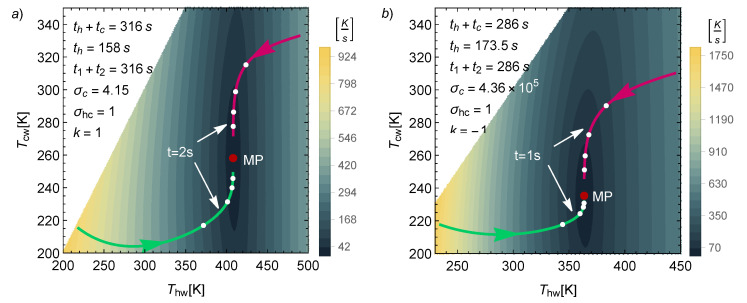
Isocontours of the relaxation velocity for (**a**) the k=1 case and (**b**) the k=−1 case. The values $T_{h}=500$ K, $\sigma_{hc}=1$, and τ=0.4 were fixed. As in Figure 2, $\sigma_{c}$ and $t_{h}$ were chosen (for representation/comparison purposes) in such a way that the MP was 70 W and the entropy at MP was 70 J/K in both cases. By fixing *S* at MP conditions, a time scale for $t_{h}$ was established, and therefore, a scale for the relaxation time. The qualitative behavior for the other values of *S* was similar to the one presented here.

From these dynamic equations, it is possible to calculate a relaxation velocity, $v=\sqrt{{\dot{T}}_{hw}^{2}+{\dot{T}}_{cw}^{2}}$ (in K/s), which indicates how fast the system is evolving toward the steady state. In Figure 3, iso-velocity contours are depicted for the k={1,−1} cases. Two trajectories are represented to provide an idea of how fast the system can return to the MP state. In both cases, the operation time is indicated, along with the time of evolution of each curve. As can be seen, 1 or 2 s are enough to drive the system close to the stationary state; meanwhile, the cycle time is approximately 300 s. Another feature in Figure 3 is that far from the steady state, the system evolves faster. Different marks at equal time intervals are placed over the trajectories, and in every case, the system travels a longer distance in the first interval. In both cases, the $T_{cw}$ direction is slower. It can be confirmed that the depicted trajectories evolve more quickly in the horizontal direction, while the final approach is mostly in the vertical direction. Notice also that the k=−1 case has the fastest dynamics.

## 5. Thermodynamics of the Relaxation Trajectories

From the analysis of the evolution of trajectories toward the steady state, it is not obvious which energetic implications arise. To address this issue, the dynamic equations were numerically solved. Several trajectories with a starting point within a radius of 30K (in the $T_{hw}$–$T_{cw}$ space) from the MP state were analyzed. Then, these trajectories were mapped into the energetic space (η, *P*, and $\dot{S}$).

As it can be checked in Equations (4), (17) and (18), the conductance ratio $\sigma_{hc}$ plays a key role both in the dynamic equations and in the energetic properties of the system. In this regard, it is noted that the limits $\sigma_{hc}\to \left\{0,\infty \right\}$ are not physical from a dynamic point of view. For both the k={−1,1} cases, the limit $\sigma_{hc}\to 0$ leads to null power output *P*, entropy *S* (Equation (5)), and also null restitution strengths (see Equation (18)), i.e., {P,S,A,B}→0; thus, there is no power output and there are no restitution forces. In comparison, the limit $\sigma_{hc}\to \infty$ leads to {S,A,B}→∞; thus, there is an infinite entropy generation and infinite restitution forces. For this reason, in the present analysis, the more realistic cases $\sigma_{hc}=\left\{10^{-6},1,10^{6}\right\}$ were considered.

**Figure 4:**
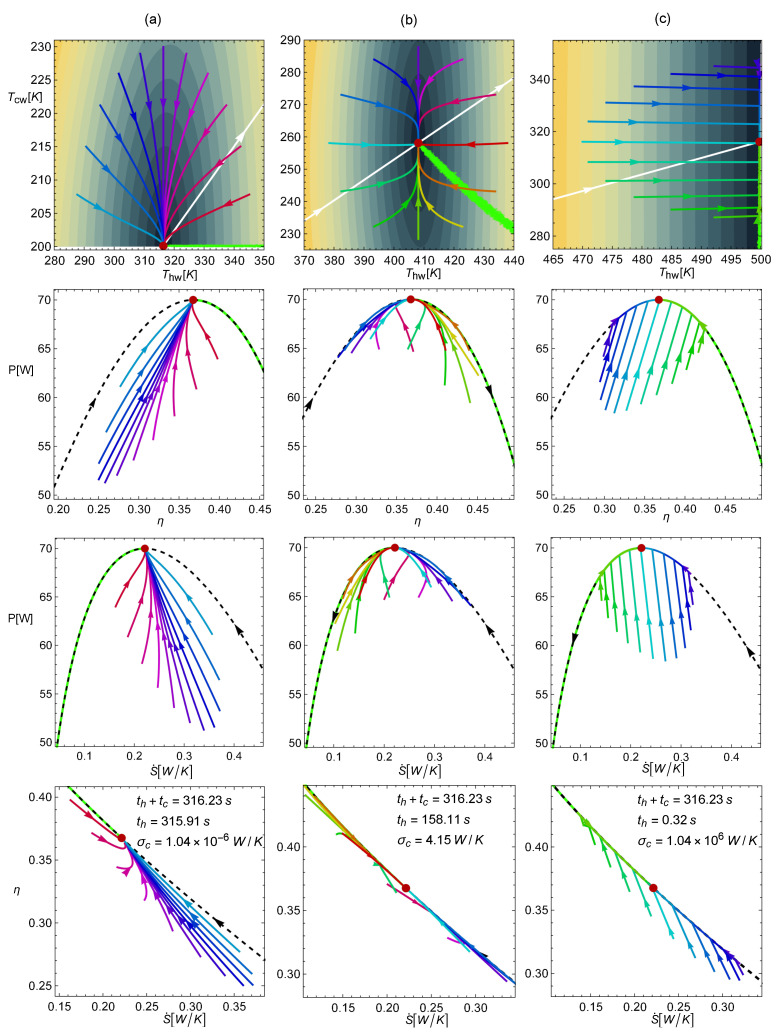
Trajectories in the $T_{hw}$–$T_{cw}$ space and the mapping over the η–*P*, $\dot{S}$–*P*, and $\dot{S}$–η spaces for k=1. (**a**) the $\sigma_{hc}=10^{-6}$ case; (**b**) the $\sigma_{hc}=1$ case; (**c**) the $\sigma_{hc}=10^{6}$ case. The values $T_{h}=500$ K and τ=0.4 were fixed. As in Figure 2, $\sigma_{c}$ and $t_{h}$ were chosen (for representation/comparison purposes) in such a way that the MP was 70 W and the entropy at MP was 70 J/K in both cases.

In Figure 4, the four rows display the trajectories obtained from numerically solving the dynamics in Equation (18) for the k=1 case. The first row displays the trajectories in the $T_{hw}$ and $T_{cw}$ space, while the next rows show, respectively, the mapping of the same trajectories over the η–*P*, $\dot{S}$–*P*, and $\dot{S}$–η spaces. The three columns are for the abovementioned representative conductance values: (a) $\sigma_{hc}=10^{-6}$, (b) $\sigma_{hc}=1$, and (c) $\sigma_{hc}=10^{6}$. In the first row, according to the initial state in each trajectory, a clock hour analogy was used; in this way, the purple line corresponds to 12 o’clock and red line to 3 o’clock. The Pareto optimal set is displayed (green points), together with some iso-velocity contours. The white curve indicates the position of the MP state as $\sigma_{hc}$ varies from 0 to *∞*. All of these points produce the same efficiency at MP given by $\eta^{*}=\eta_{CAN}$. For the rest of the rows, the Pareto front and the endoreversible curve are presented as well. Note also that in the three configurations (columns), the total operation time is the same, but the $t_{c}/t_{h}$ (and $\sigma_{c}$) ratio varies, as can be seen in the legend in each case. This figure reveals several key features:

(a) For a small $\sigma_{hc}$ (left column), only trajectories between 9 and 3 o’clock are present in the $T_{hc}$–$T_{cw}$ space (denoted in colors ranging from red to blue). Most of the observed trajectories (especially those with darker colors) evolve to the stable state near the endoreversible curve in such a way that the power and efficiency progressively increase their values, while entropy production decreases (see rows 2–4 in the first column). In other words, the relaxation process mainly drives the system toward a thermodynamic steady state, thereby enhancing the thermodynamic performance of the engine (η and *P* increase and $\dot{S}$ decreases).

(b) For $\sigma_{hc}=1$ (second column), the trajectories arriving at the stable point from all directions are clearly observed. Those in darker colors (from 9 to 3 o’clock) evolve, as in the above case, but closer to the endoreversible curve, showing an increase in power and efficiency and a decrease in entropy generation. In contrast, the trajectories denoted in colors ranging from orange to green (between 3 and 7 o’clock) evolve to the steady state near the Pareto front, with an increase in power output but a decrease in efficiency and higher entropy production. Note also in this configuration how the relaxation is well balanced between the trajectories approaching the endoreversible limit on the Pareto front side and to the other side. However, the curves near the Pareto front are longer, meaning that random perturbations tend to favor a locus directed toward the Pareto front.

(c) For a large $\sigma_{hc}$ (last column), the trajectories evolve directly toward the endoreversible curve first. In this part of the trajectory, η, *P*, and $\dot{S}$ are enhanced simultaneously. Later on, the system evolves to the steady state, either through the endoreversible limit or the Pareto front.

(d) Although the sizes of the perturbations in the $T_{hw}$–$T_{cw}$ space are the same, the case with the small $\sigma_{hc}$ allows larger variations of power output, while the case where $\sigma_{hc}=1$ exhibits the smallest fluctuations in the η–$\dot{S}$ plane.

(e) The relaxation times (Equation (17)) are directly proportional to $t_{h}$, and from the expressions of *P*, η, and *S* (see Equation (5)), only entropy generation depends on $t_{h}$. Thus, the characteristic time scale of the relaxation is linked to the entropy scale of the system, a feature that connects the stability with the thermodynamics of the system. In this entropy–control point of view, the dynamics for the small values of $\sigma_{hc}$ in the left column apply to greater $t_{h}$ values and, as a consequence, when the contact time of the heat engine with the cold reservoir is small. On the contrary, the dynamics for the large values of $\sigma_{hc}$ in the right column apply to smaller $t_{h}$ values, i.e., when the contact time of the heat engine with the cold reservoir is large.

**Figure 5:**
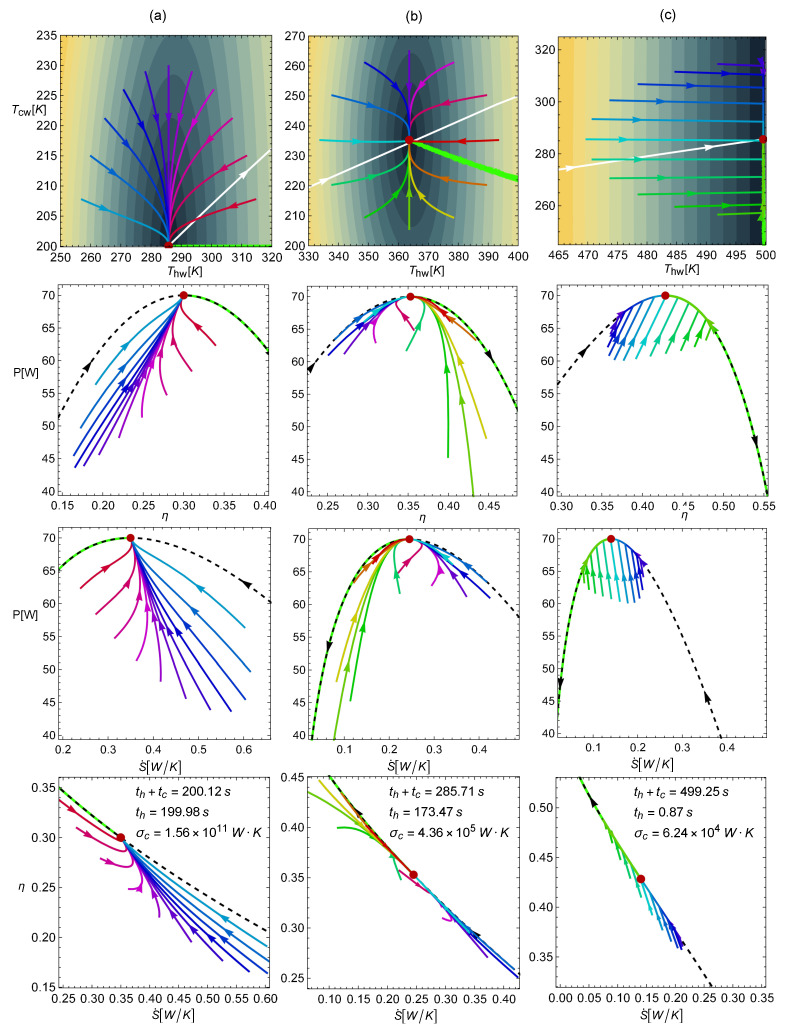
Trajectories in the $T_{hw}$–$T_{cw}$ space and mapping over the η–*P*, $\dot{S}$–*P*, and $\dot{S}$–η spaces for k=−1. In column (**a**) the $\sigma_{hc}=10^{-6}$ case; (**b**) the $\sigma_{hc}=1$ case; (**c**) the $\sigma_{hc}=10^{6}$ case. The values $T_{h}=500$ K and τ=0.4 were fixed. As in Figure 2, $\sigma_{c}$ and $t_{h}$ were chosen (for representation/comparison purposes) in such a way that the MP was 70 W and the entropy at MP was 70 J/K in both cases.

In Figure 5, the corresponding configurations for k=−1 are depicted. Notice that the behavior of the trajectories is almost the same as that in the k=1 case, but here, the maximum power stationary state is linked to different efficiencies as $\sigma_{hc}$ increases from 0 to *∞*. In particular, when $\sigma_{hc}\to 0$, the efficiency at maximum power is $\eta^{*}=\frac{1-\tau }{2}=\frac{\eta_{c}}{2}$; when $\sigma_{hc}\to 1$, $\eta^{*}=\frac{2(1-\tau )}{3+\tau }=\frac{2\eta_{C}}{4-\eta_{C}}$; when $\sigma_{hc}\to \infty$, $\eta^{*}=\frac{1-\tau }{1+\tau }=\frac{\eta_{C}}{2-\eta_{C}}$. The dynamics for different $\sigma_{hc}$ are no longer linked to equal operation times $t_{c}+t_{h}$. Another difference is that the relaxation times are noticeably smaller and, therefore, the restitution forces are stronger. Additionally, as $\sigma_{hc}$ decreases ($\eta^{*}$ also decreases), the same temperature perturbations lead to the farthest starting points in the energetic planes. In this way, the case of tlarge $\sigma_{hc}$ (with a high efficiency) is the one with the smallest drops in η, *P*, and $\dot{S}$ (with shorter trajectories). This suggests that the system becomes more stable as the efficiency in the steady state increases.

## 6. Concluding Remarks

It was shown that the Pareto front, which represents the best compromise among power output, efficiency, and entropy generation is related to endoreversible behavior, obtained herein by analyzing the Newton and phenomenological heat transfer laws in the context of Carnot-like models. These findings corroborate those obtained in the low-dissipation scheme.

A set of dynamic equations were found based only on the assumption that the endoreversible heat engine has a stationary state and from a Taylor expansion of the input/output heat fluxes in the first order. A relationship between relaxation times and total operation time was obtained. It was shown that the trajectories that lead the system back to the stationary state require much shorter times than the cycle time, allowing the system to work under continuous cyclic operation.

Mapping of the relaxation trajectories into the energetic space allowed for an analysis of the performance consequences of stability. The degree of symmetry of the conductance ratio in the input/output heat exchange is the main valuable factor for stability dynamics. For small $\sigma_{hc}$ values, the thermodynamic trajectories improve the efficiency and power values with a decrease in entropy generation, evolving near the endoreversible behavior. For larger $\sigma_{hc}$ values, the first part of the thermodynamic trajectories improve the efficiency and power values with a decrease in entropy generation, evolving toward the endoreversible behavior and the Pareto front. In between these two situations, i.e., for equal conductance values, ($\sigma_{hc}=1)$ trajectories with decreasing efficiencies and increasing entropy generation can be found, with a preference for evolving near the Pareto front.

In summation, the stability of the irreversible Carnot-like heat engine exhibits two interesting behaviors in which the fluctuations around the stationary state (due to external perturbations) would likely maintain the system in an optimum state, or produce self-optimization induced by the stability. A biased control of the operation parameters could result in an economic saving, as energy is needed for the fine-tuning of parameter control. Finally, in this work the thermodynamic functions that were selected as the most relevant for this heat engine model were η, *P*, and $\dot{S}$. It is very likely that by analyzing different stationary states, such as those predicted by the compromise Ecological [41] or Omega [42] operation performances, the role of the Pareto front would be more evident, as occurred in the case of the low-dissipation heat engine and refrigerator [1,2].

Another valuable remark is that the total entropy generation defines a time scale for both the operation and relaxation times. Finally, this analysis laid down the grounds to analyze heat devices dependent on working fluid properties, where the endoreversible hypothesis plays a relevant role. Especially when the thermalization mechanisms are fast enough compared to the cycle time in agreement with local equilibrium. This allows to incorporate, in a straightforward way, stochastic-type perturbations into the analysis through additive noise in Equation (14). 

## References

[1] Gonzalez-Ayala J., Guo J., Medina A., Roco J.M.M., Hernández A.C. (2020). Energetic Self-Optimization Induced by Stability in Low-Dissipation Heat Engines. Phys. Rev. Lett..

[2] Gonzalez-Ayala J., Medina A., Roco J.M.M., Hernández A.C. (2020). Thermodynamic optimization subsumed in stability phenomena. Sci. Rep..

[3] Gonzalez-Ayala J., Guo J., Medina A., Roco J.M.M., Hernández A.C. (2019). Optimization induced by stability and the role of limited control near a steady state. Phys. Rev. E.

[4] Esposito M., Kawai R., Lindenberg K., van den Broeck C. (2010). Efficiency at maximum power of low-dissipation Carnot engines. Phys. Rev. Lett..

[5] Curzon F., Ahlborn B. (1975). Efficiency of a Carnot engine at maximum power output. Am. J. Phys..

[6] Berry R.S., Salamon P., Andresen B. (2020). How It All Began. Entropy.

[7] Gonzalez-Ayala J., Roco J.M.M., Medina A., Hernández A.C. (2017). Carnot-like heat engines versus low-dissipation models. Entropy.

[8] Johal R.S. (2017). Heat engines at optimal power: Low-dissipation versus endoreversible model. Phys. Rev. E.

[9] Gonzalez-Ayala J., Medina A., Roco J.M.M., Hernández A.C. (2018). Entropy generation and unified optimization of Carnot-like and low-dissipation refrigerators. Phys. Rev. E.

[10] Santillán M., Maya G., Angulo-Brown F. (2001). Local stability analysis of an endoreversible Curzon-Ahborn-Novikov engine working in a maximum-power-like regime. J. Phys. D.

[11] Guzmán-Vargas L., Reyes-Ramírez I., Sánchez N. (2005). The effect of heat transfer laws and thermal conductances on the local stability of an endoreversible heat engine. J. Phys. D.

[12] Chimal-Eguia J., Guzmán-Vargas L., Reyes-Ramírez I. (2007). Local Stability of an Endoreversible Heat Engine Working in an Ecological Regime. Open Syst. Inf. Dyn..

[13] Huang Y., Sun D., Kang Y. (2009). Local stability characteristics of a non-endoreversible heat engine working in the optimum region. Appl. Therm. Eng..

[14] Ladino-Luna D., Portillo-Díaz P., Páez-Hernández R.T. (2013). Local Stability of Curzon-Ahlborn Cycle with Non-Linear Heat Transfer for Maximum Power Output Regime. J. Mod. Phys..

[15] Reyes-Ramírez I., Barranco-Jiménez M., Rojas-Pacheco A., Guzmán-Vargas L. (2014). Global Stability Analysis of a Curzon–Ahlborn Heat Engine under Different Regimes of Performance. Entropy.

[16] Reyes-Ramírez I., Barranco-Jiménez M., Rojas-Pacheco A., Guzmán-Vargas L. (2014). Global stability analysis of a Curzon–Ahlborn heat engine using the Lyapunov method. Physica A.

[17] Chen L., Wu X., Xiao Q., Ge Y., Sun F. (2018). Local stability of a generalized irreversible heat engine with linear phenomenological heat transfer law working in an ecological regime. Ther. Sci. Eng. Prog..

[18] Valencia-Ortega G., Levario-Medina S., Barranco-Jiménez M.A. (2020). Local and global stability analysis of a Curzon-Ahlborn model applied to power plants working at maximum *κ*-efficient power. arXiv.

[19] Barranco-Jiménez M., Sánchez-Salas N., Reyes-Ramírez I. (2015). Local Stability Analysis for a Thermo-Economic Irreversible Heat Engine Model under Different Performance Regimes. Entropy.

[20] Huang Y., Sun D., Kang Y. (2007). Local stability analysis of a class of endoreversible heat pumps. J. Appl. Phys..

[21] Huang Y., Sun D. (2008). The effect of cooling load and thermal conductance on the local stability of an endoreversible refrigeratorImpact de la charge thermique et de la conductivité thermique sur la stabilité locale d’un réfrigérateur endoréversible. Int. J. Refrig..

[22] Nie W., He J., Yang B., Qian X. (2008). Local stability analysis of an irreversible heat engine working in the maximum power output and the maximum efficiency. Appl. Therm. Eng..

[23] Nie W., He J., Deng X. (2008). Local stability analysis of an irreversible Carnot heat engine. Int. J. Therm. Sci..

[24] Huang Y. (2009). Local asymptotic stability of an irreversible heat pump subject to total thermal conductance constraint. Energy Convers. Manag..

[25] He J., Miao G., Nie W. (2010). Local stability analysis of an endoreversible Carnot refrigerator. Phys. Scr..

[26] Wouagfack P.A.N., Keune G.F., Tchinda R. (2017). Local stability analysis of an irreversible refrigerator working at the maximum thermo-ecological functions: A comparison. Analyse de la stabilité locale d’un réfrigérateur irréversible fonctionnant au maximum de ses fonctions thermo-écologiques: Une comparaison. Int. J. Refrig..

[27] Lü K., Nie W., He J. (2018). Dynamic robustness of endoreversible Carnot refrigerator working in the maximum performance per cycle time. Sci. Rep..

[28] Sekulic D.P. (1998). A fallacious argument in the finite time thermodynamics concept of endoreversibility. J. Appl. Phys..

[29] Andresen B. (2001). Comment on “A fallacious argument in the finite time thermodynamic concept of endoreversibility” [J. Appl. Phys. 83, 4561 (1998)]. J. Appl. Phys..

[30] Chen J., Yan Z., Lin G., Andresen B. (2001). On the Curzon–Ahlborn efficiency and its connection with the efficiencies of real heat engines. Energy Convers. Manag..

[31] Gyftopoulos E.P. (2002). On the Curzon–Ahlborn efficiency and its lack of connection to power producing processes. Energy Convers. Manag..

[32] Rojas-Gamboa D.A., Rodríguez J.I., Gonzalez-Ayala J., Angulo-Brown F. (2018). Ecological efficiency of finite-time thermodynamics: A molecular dynamics study. Phys. Rev. E.

[33] Izumida Y., Okuda K. (2008). Molecular kinetic analysis of a finite-time Carnot cycle. EPL.

[34] Izumida Y., Okuda K. (2017). Molecular kinetic analysis of a local equilibrium Carnot cycle. Phys. Rev. E.

[35] Muschik W., Hoffmann K.H. (2020). Modeling, Simulation, and Reconstruction of 2-Reservoir Heat-to-Power Processes in Finite-Time Thermodynamics. Entropy.

[36] Agrawal D.C., Gordon J.M., Huleihil M. (1994). Endoreversible engines with finite-time adiabats. Indian J. Eng. Mater. Sci..

[37] Hernandez A.C., Roco J.M.M., Medina A., Velasco S., Guzmán-Vargas L. (2015). The maximum power effciency 1−$\sqrt{\tau }$: Research, education, and bibliometric relevance. Eur. Phys. J. Spec. Top..

[38] Deb K. (2001). Multi-Objective Optimization Using Evolutionary Algorithms.

[39] Kullback S., Leibler R.A. (1951). On information and sufficiency. Ann. Math. Stat..

[40] Venables W.N., Ripley B.D. (2002). Modern Applied Statistics with S.

[41] Angulo-Brown F. (1991). An ecological optimization criterion for finite-time heat engines. J. Appl. Phys..

[42] Hernández A.C., Medina A., Roco J.M.M., White J.A., Velasco S. (2001). Unified optimization criterion for energy converters. Phys. Rev. E.

[43] Strogatz S.H. (2014). Nonlinear Dynamics and Chaos: With Applications to Physics, Biology, Chemistry, and Engineering.

